# Herpes simplex virus assembly and spread in murine skin after infection from the outside

**DOI:** 10.1128/jvi.01638-24

**Published:** 2025-02-13

**Authors:** Timmy Richardo, Xiaokun Liu, Katinka Döhner, Tsung-Yu Chao, Anna Buch, Anne Binz, Anja Pohlmann, Madeleine de le Roi, Wolfgang Baumgärtner, Korbinian Brand, Rudolf Bauerfeind, Reinhold Förster, Beate Sodeik, Stephan Halle

**Affiliations:** 1 Institute of Virology, Hannover Medical School 686461 , Hannover, Germany; 2 RESIST - Cluster of Excellence, Hannover Medical School 9177 , Hannover, Germany; 3 Institute of Immunology, Hannover Medical School 9177 , Hannover, Germany; 4 Department of Pathology, University of Veterinary Medicine Hannover 26556 , Hannover, Germany; 5 Institute of Clinical Chemistry and Laboratory Medicine, Hannover Medical School 9177 , Hannover, Germany; 6 Research Core Unit Laser Microscopy, Hannover Medical School 9177 , Hannover, Germany; 7 German Center for Infection Research (DZIF), Hannover-Braunschweig Partner Site, Hannover, Germany; University of Virginia, Charlottesville, Virginia, USA

**Keywords:** skin infection, HSV-1, HSV-1 pathogenesis, virus spread

## Abstract

Herpes simplex viruses (HSV) cause many skin diseases, particularly in immunocompromised patients. HSV-1 infection of murine skin recapitulates many aspects of human pathology. However, many protocols rely on mechanical or enzymatic skin disruption to induce lesions, although this can alter skin homeostasis and prime antiviral inflammation before inoculation. To investigate the initial events following HSV-1 primary skin infection before the onset of symptoms, we developed a novel murine *ex vivo* explant model using gentle depilation without further scarification and infected keratinocytes from the outside with minimal tissue damage. Two-photon microscopy showed that HSV-1 spread exclusively in the epidermis. The infection centers increased in number and size over time and contained hundreds of infected keratinocytes. We investigated the HSV-1 spread at the cellular level, using reporter strains with fluorescently tagged capsid protein VP26, and observed the formation of nuclear capsid assembly sites and nuclear capsid egress and the recruitment of the inner tegument protein pUL37GFP, the outer tegument protein VP11/12GFP, and the envelope protein gDGFP to cytoplasmic capsids. By using electron microscopy, the skin appeared intact, and keratinocytes contained many nuclear capsids, primary virions in the nuclear envelope, cytosolic membrane-associated capsids, and enveloped virions. Our protocol provides a robust and reproducible approach to investigate the very early events of HSV-1 spread in the skin, to characterize the phenotypes of HSV-1 mutants in terminally differentiated skin tissues, and to evaluate potentially antiviral small molecules in a preclinical *ex vivo* infection model.

**IMPORTANCE:**

This study describes a novel murine *ex vivo* skin explant model to investigate early events in HSV-1 infection without causing significant tissue damage. To infect from the outside, via the apical keratinocytes, this method relies on gentle depilation, which maintains skin integrity. HSV-1 spread exclusively within the epidermis, with infection centers increasing over time and involving hundreds of keratinocytes. Using advanced microscopy techniques, we tracked HSV-1 spread at the cellular level and intracellular assembly of all intermediate virus structures. This model offers a valuable tool for studying the initial stages of HSV-1 infection, assessing viral mutant phenotypes, and testing antiviral compounds in a more physiological context to provide critical insights into HSV-1 pathogenesis and therapeutic strategies.

## INTRODUCTION

The World Health Organization (WHO) estimates that about two-thirds of the human population is infected with herpes simplex virus (HSV) type 1 and about 13% with HSV-2 (1, 2). HSV-1 typically causes cold sores and lesions on the oral mucosa and the perioral skin, while HSV-2 can lead to genital skin lesions and ulcers (3, 4). After infection from the outside and local replication, HSV-1 and HSV-2 enter neurites by innervating the primary site of inoculation and establish lifelong latent infections in the neurons of the cranial and dorsal root ganglia (5, 6). Upon reactivation in these latently infected neurons, progeny HSV particles return via anterograde transport and re-infect the skin or the mucosa from the inside.

Primary HSV-1 infections, as well as reactivations from latency, can lead to fatal encephalitis, blinding keratitis, disseminated disease, or *eczema herpeticum* (4, 7–10). About 22% of patients with moderate-to-severe atopic dermatitis have a history of *eczema herpeticum* (10–12). Moreover, the stigma associated with facial or genital lesions leads to psychological distress. Despite the availability of life-saving antiviral drugs like acyclovir, current treatments are still inadequate, in particular for immunocompromised patients as well as for the very young and the elderly; up to 70% of the patients surviving herpes simplex encephalitis maintain neurological symptoms (7, 13).

Healthy skin provides an effective barrier against pathogens (14–16). The epidermis is a tight epithelium composed of the apical *stratum corneum*, the *stratum granulosum*, the *stratum spinosum*, and the *stratum basale*. It consists mainly of keratinocytes, which also line up the hair follicles, but also entails melanocytes, macrophages, Langerhans cells, and T cells. The thicker dermis underneath the epidermis consists mainly of fibroblasts and the extracellular collagen matrix and contains blood and lymphatic vessels. Nevertheless, HSV can infect the skin, and its replication in keratinocytes and fibroblasts leads to the development of herpetic lesions (17–21).

HSV-1 infects abdominal skin explants from healthy donors *ex vivo* after removal of the dermis and the basement membrane from the basolateral side, and *ex vivo* infection of human oral epithelial cells is also productive (22, 23). Moreover, mechanical wounding with a cosmetic derma roller is sufficient to disrupt the protective skin barrier against HSV-1 infection, to bypass the *stratum corneum* from the apical surface, and to infect the keratinocytes (24, 25). Upon *ex vivo* infection of inner human genital foreskin biopsies from infants or adults, HSV-1 infects predominantly epidermal keratinocytes and mucosal epithelial cells but has little access to dermal fibroblasts, while *ex vivo* infection of isolated fibroblasts yields high titers of viral progeny (26, 27). Infection of human inner foreskin is also possible with high-density microarray projections when the microneedles pre-coated with HSV-1 penetrate the epidermis beyond one-third of its thickness (27).

Although the anatomy and immunology of human and murine skin differ (15), infection of murine skin recapitulates many aspects of human HSV-1 skin diseases (28–31). Inoculation of mice after flank scarification results in local HSV-1 amplification and the formation of primary lesions that heal within approximately 1 week (32–34). In such experiments, the skin homeostasis might be altered, and inflammatory responses are activated before the infection as the epidermis and dermis are often dissociated to some extent by proteases, abrasion, or hair removal by waxing or tape-stripping (34–37). These models have, nevertheless, provided important insights into the immune responses in the skin (20, 38–40). Moreover, HSV-1 spread has been studied *ex vivo* in murine skin explants, but most studies have focused on infecting the dermis, which recapitulates skin infection from the inside after reactivation from latency (41–45).

Since the very early events of skin lesion formation before the onset of symptoms are not well understood, we have established an experimental system to investigate the initiation of HSV-1 infection from the outside in terminally differentiated murine skin explants. Using confocal and two-photon fluorescence microscopy, we show that HSV-1 had formed infection centers already at 12 hours post-inoculation and then spread laterally in terminally differentiated cells. Our HSV-1 dual-color reporter strains HSV1-CheVP26-pUL37GFP and HSV1-CheVP26-VP11/12GFP faithfully reflected the sequential acquisition of inner and outer tegument proteins onto capsids. Using those reporter strains with fluorescent tags on the capsid, the tegument, and the viral envelope, as well as electron microscopy, we show that all HSV-1 assembly intermediates were formed and that capsids underwent primary envelopment, recruited the inner tegument protein pUL37 and the outer tegument protein VP11/12 in the cytoplasm, and underwent secondary envelopment on cytoplasmic membranes containing the envelope glycoprotein gD. Our protocol using skin explant cultures bridges studies infecting primary cells cultured on plastic or on glass with animal infection experiments. It provides a versatile, cost-effective system to investigate cutaneous HSV-1 infection, to characterize the phenotype of HSV-1 mutants, and to test the potency of novel small-molecule candidates for antiviral therapy, which likely could be expanded to other cutaneous viral and bacterial infections.

## RESULTS

### A novel *ex vivo* model for HSV-1 skin infection

**Fig 1:**
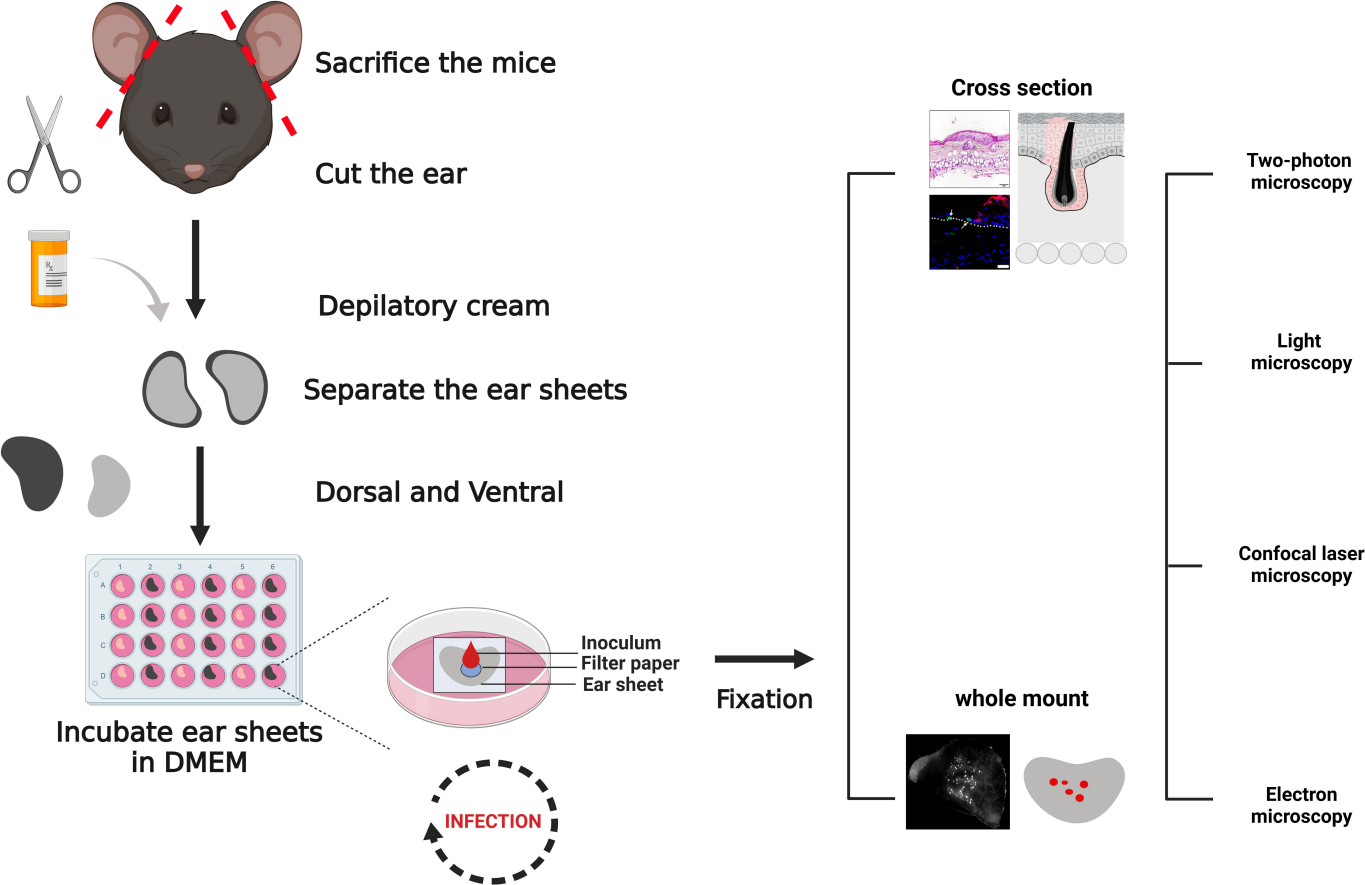
*Ex vivo* HSV-1 infection of murine skin. The ears of sacrificed mice were collected and depilated. The entire dorsal and ventral ear sheets of about 1 cm^2^ were placed on a nylon gaze in an air–liquid interface culture with the dermis side facing the medium. Filter papers with a diameter of 5 mm were positioned onto the epidermis surface, and 10 µL of buffer without or with HSV-1 was pipetted directly onto a given filter paper. After an incubation of 30 minutes at 37°C, the filter papers were removed, and the explants were cultured at 37°C for up to 96 hours, fixed or lysed, and processed for microscopy, molecular biology, or virology assays. Semi-thin cryosections and paraffin-sections were labeled with primary antibodies against host or HSV-1 proteins, followed by secondary fluorescent antibodies. HSV-1-infected cells were localized also by the expression of fluorescent proteins of HSV-1 reporter strains. The specimens were analyzed by confocal or two-photon fluorescence microscopy as well as by electron microscopy. Created with BioRender.com.

**Fig 2:**
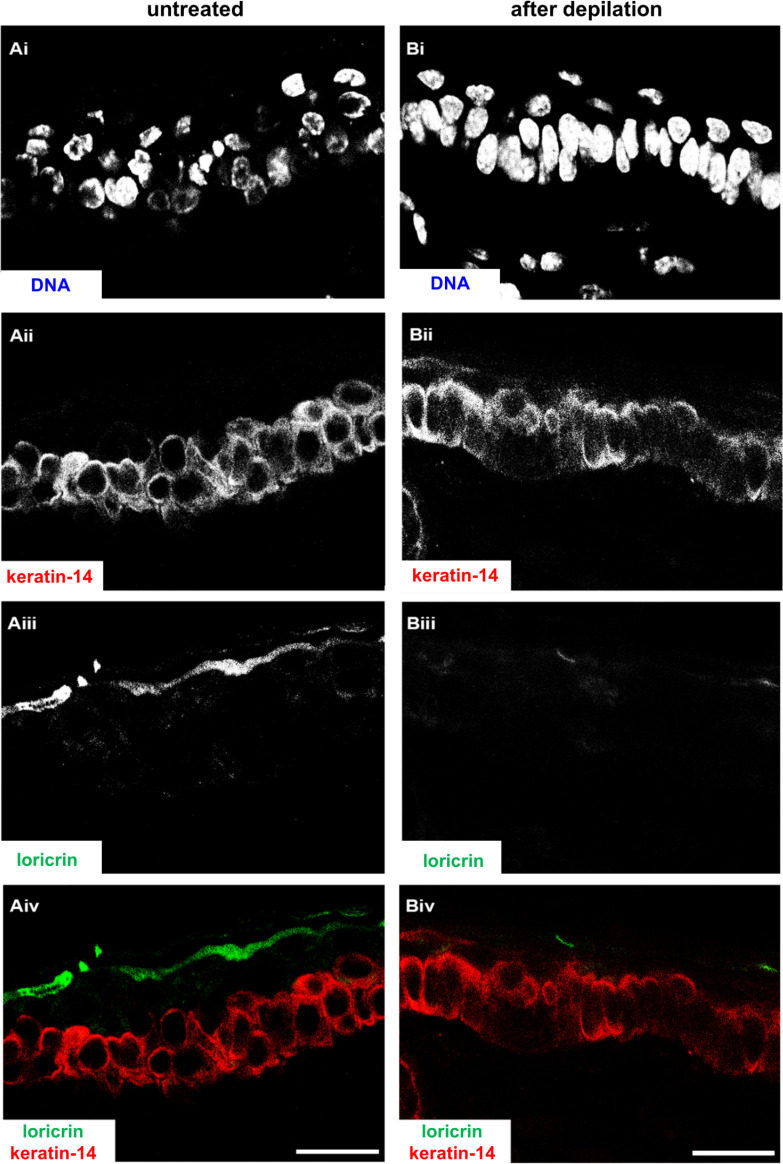
Depilation-induced changes in skin morphology. Uninfected murine ear sheets without (**A**) or after (**B**) depilation were cultured at the air–liquid interface for 48 hours, fixed, and embedded. Cryosections were stained with DAPI (DNA; Ai, Bi), labeled for keratin-14 (Aii, Bii red in Aiv, Biv) and loricrin (Aiii, Biii green in Aiv, Biv), and analyzed by confocal fluorescence microscopy. Scale bar: 25 µm.

To investigate the very early events of HSV-1 skin infection, we used explant cultures from the ears of 6- to 9-week-old mice. The hair was removed by a short, 5-minute treatment with a depilatory cream (Fig. 1). The ears were then split into dorsal and ventral sheets and glued onto slightly larger tissue gaze pieces, which in turn were placed onto the cell culture medium to generate an air–liquid interface culture. Immunohistochemistry analyses of cross-sections showed that the depilation had not removed basal keratinocytes expressing keratin-14-expressing keratinocytes (Fig. 2Aii and Bii red in Aiv, Biv), but reduced the labeling for loricrin, and possibly the amount of some apical keratinocytes (Fig. 2Aiii and Biii; green in Aiv, Biv). Together with keratin 1 and keratin 10, loricrin is a major protein of the cornified keratinocytes (46, 47).

**Fig 3:**
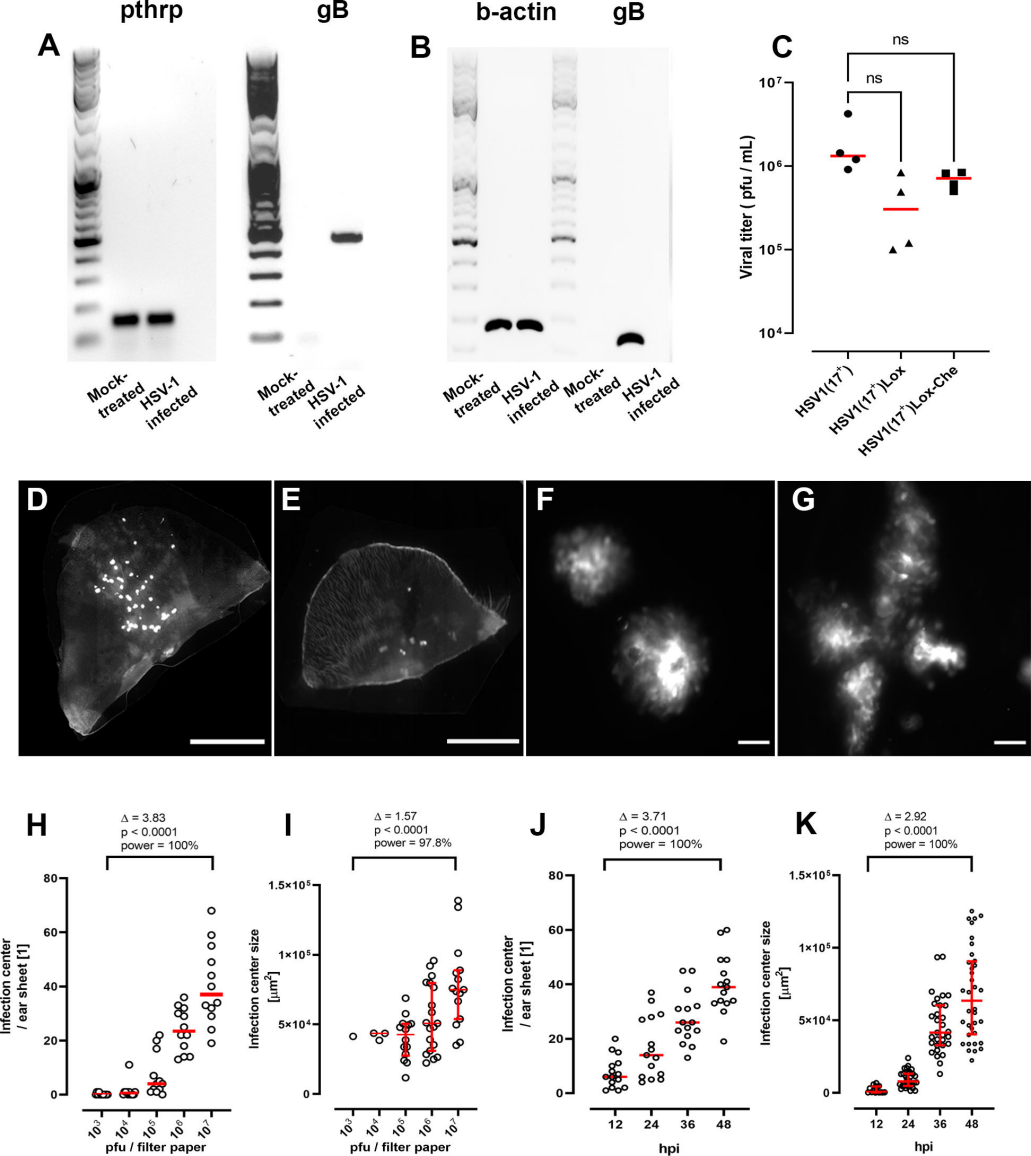
*Ex vivo* infection of murine skin sheets with HSV-1. Murine ear sheets were mock-treated or infected with HSV1(17^+^)Lox-Che (**A–K**), HSV1(17^+^)Lox (**C**), or HSV1(17^+^) (**C**) at 1 × 10^7^ (**A–G, J, K**) or at 10^3^ to 10^7^ (**H, I**) pfu/filter paper for 48 hours (**A–I**) or 12 to 48 hours (**J, K**). (**A**) At 48 hpi, the DNA was isolated from the explants, and the amount of murine genomes was determined by PCR for parathyroid hormone-related protein (pthrp) and the amount of HSV-1 genomes by PCR for UL27 encoded gB. (**B**) At 48 hpi, the RNA was isolated, and the amount of murine transcripts was determined by RT-PCR for β-actin and the amount of HSV-1 transcripts by PCR for gB. (**C**) At 48 hpi, the amount of infectious HSV-1 was determined by plaque assay in four independent biological replicates. (D–G) Ear sheets inoculated after depilation (**D, F, G**) or after mock-treatment without depilatory cream (**E**) were fixed, and the infectious centers, as indicated by the HSV-1 Che expression in whole-mounts, were analyzed by fluorescence microscopy at low (D, E; scale bar: 500 µm) or high magnification (F, G; scale bar: 50 µm). (H–K) Ear sheets were infected with increasing amounts of HSV1-Che for 48 hours (**H, I**) or at 1 × 10^7^ pfu/filter paper for 12 to 48 hours (**J, K**), fixed, and the number (**H, J**) and the size (**I, K**) of the infection centers were determined. Mean (H–K; red) with standard error of the mean (I, K; red), effect size (Δ), *P*-value, and *post-hoc* statistical power are indicated from the compiled data of three independent biological replicates.

For virus inoculation, round filter papers were placed onto the center of a given dorsal or ventral skin sheet, and a medium containing HSV1(17^+^)Lox-Che was added directly onto the filter papers (Fig. 1). This reporter strain expresses fluorescent mCherry under the control of a constitutively active viral promoter (48). After 30 minutes, the filter papers were removed, and the explants were transferred back to a 37°C incubator for up to 96 hours. Within 48 hours post-infection (hpi), HSV-1 genomes (Fig. 3A) and HSV-1 transcripts (Fig. 3B) were clearly detected in infected samples. The HSV-1 gene UL27, encoding glycoprotein B, is expressed after viral replication with γ_1_ late kinetics (49). HSV1-Che is a recombinant strain derived from the bacterial artificial chromosome (BAC) pHSV1(17^+^)-Lox of the clinical isolate strain 17^+^ and, like all BAC-derived HSV-1 strains, lacks one of the three viral origins-of-replication (50). As BAC-derived HSV-1 strains are impaired in some animal infection models, in particular in reactivation from latency (51), we compared the infection of the BAC-derived HSV1(17^+^)Lox-Che and HSV1(17^+^)Lox strains to a low-passage preparation of HSV-1 strain 17^+^, their parental clinical isolate. The clinical isolate had replicated to slightly higher titers when compared to HSV1(17^+^)Lox-Che or HSV1(17^+^)Lox in the skin explants (Fig. 3C).

Fluorescence microscopy of whole-mount explants revealed several infection centers expressing HSV-1-encoded mCherry in the regions that had been inoculated via the filter papers but not in the surrounding skin (Fig. 3D). Without depilation, HSV1-Che rarely established infection centers, and their numbers varied stochastically among explants (Fig. 3E). Following infection using the HSV-1-soaked filter papers, the size of the infection centers was heterogeneous, and a closer inspection at higher magnification suggested that some had originated from a single focus and thus possibly from a single infected cell (Fig. 3F), while others contained multiple foci that had coalesced into a larger infected region (Fig. 3G). All skin explants contained infection centers at a dose of 10^6^ PFU/filter or more (Fig. 3H). At 48 hpi, at a dose of 10^4^ to 10^6^ PFU/filter, the median sizes of the infection centers were rather similar but larger at 10^7^ PFU/filter (Fig. 3I). At 10^7^ PFU/filter, small fluorescent infection centers had formed already within 12 hpi, and their number (Fig. 3J) and size (Fig. 3K) increased over time.

With this novel protocol, we could reproducibly inoculate with a higher virus dose than by pipetting the inoculum directly onto the skin. The increasing number of infection centers, either with a higher dose or prolonged infection time, might indicate HSV-1 spread from primary infection centers and the formation of secondary infection centers or a heterogeneous expression of intrinsic antiviral proteins. In contrast to conventional plaque assays with cell monolayers (52), we did not include any agents such as neutralizing antibodies, methylcellulose, or agarose. However, we assume that there was only lateral cell-to-cell spread in this air–liquid interface culture. Our data are consistent with the notion that increasing the dose or the infection time augmented the likelihood of forming large coalescent infection centers derived from several small infection centers.

### HSV-1 spreads in the murine epidermis but not to the dermis

**Fig 4:**
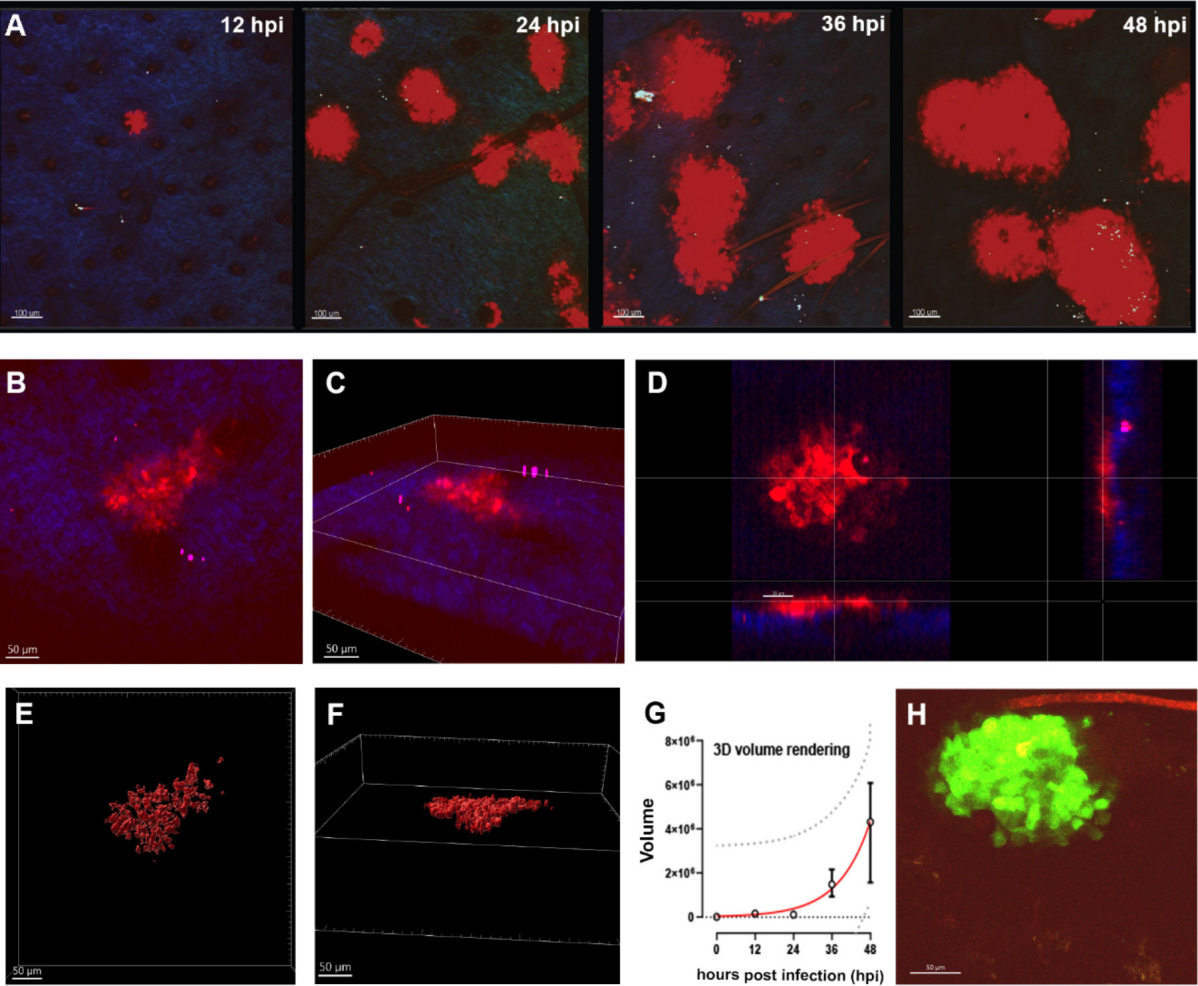
HSV-1 spreads in the epidermis but not the dermis of murine skin explants. (**A–H**) Ear sheets were infected with HSV1-Che or HSV1-GFP at 1 × 10^7^ pfu/filter paper, fixed, and analyzed by two-photon microscopy for HSV1-Che (A–G; red) or HSV1-GFP (H; green) expression and with the second harmonic signal for collagen (A–D; blue). Expansion of HSV1-Che infection centers over time (**A**). Views of infectious centers in two-dimensional (**B**), three-dimensional (**C and D**), or with rendered nuclei (**E and F**) at 48 hpi. The volume of the infection centers was measured until 48 hpi based on the rendered nuclei from two independent experiments (G; median, IQR; red line, fitted exponential growth curve; 95% prediction bands, dotted line).

Next, we characterized the organization of the infection centers by two-photon fluorescence microscopy. The infection centers were detected as early as 12 hpi with HSV1-Che (Fig. 4A). Two-dimensional (Fig. 4B) and three-dimensional (Fig. 4C and D) reconstructions indicated that the infection centers were limited to the epidermis and had expanded more in the x–y than in the z directions. The volume of the infectious centers, as measured from the rendered surface of HSV1-Che-expressing tissue (Fig. 4E and F), increased exponentially over time (Fig. 4G). Infection with HSV1-GFP, which expresses soluble monomeric GFP as a reporter, yielded centers of similar size with similar kinetics (Fig. 4H).

**Fig 5:**
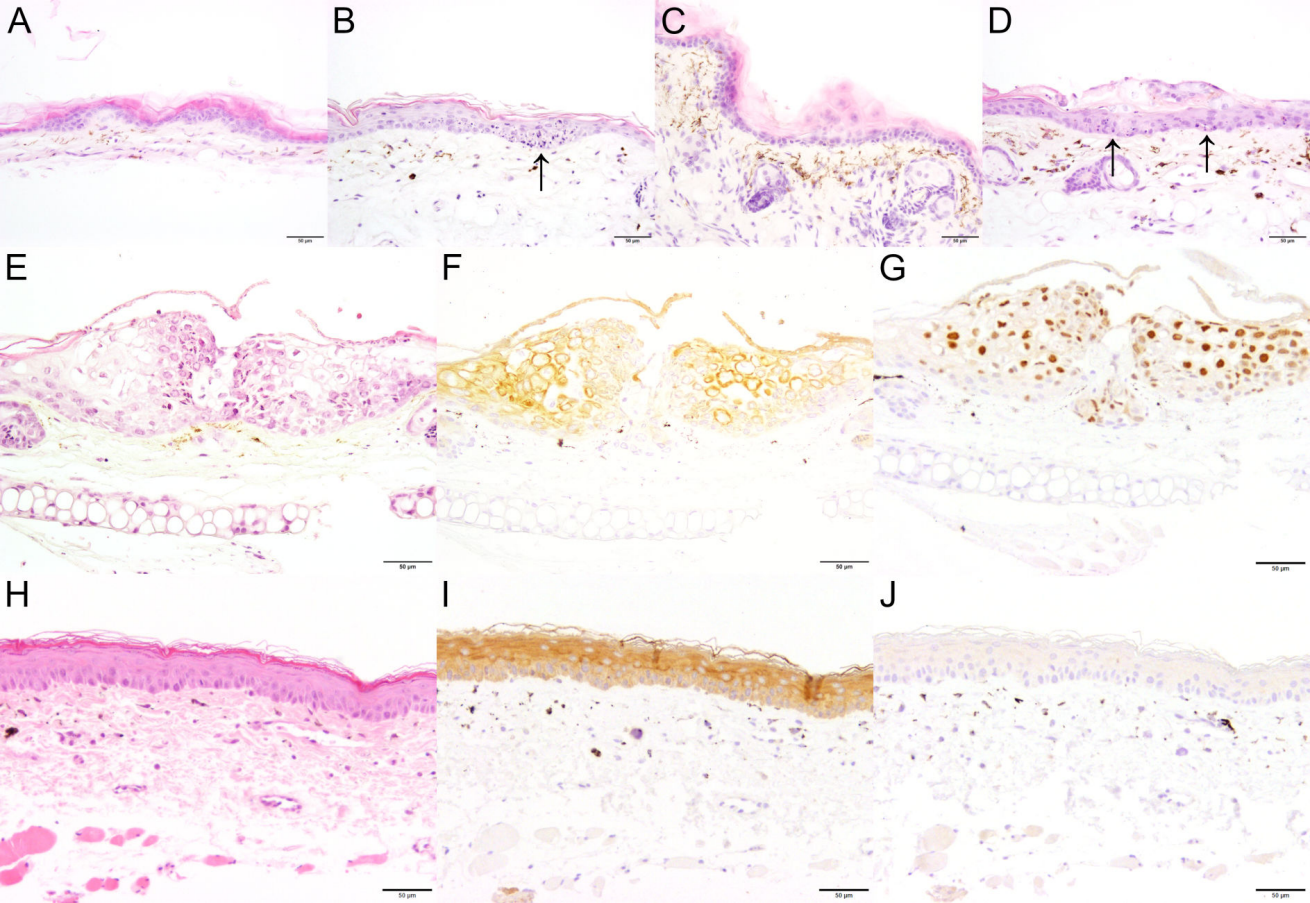
Histopathological characterization of uninfected and infected murine skin explants. Ear sheets were left untreated (**A, B**) or treated with depilatory cream (**C, D**) and processed immediately (**A, C**), or cultured *ex vivo* for 48 hours (**B, D**), and stained with hematoxylin and eosin (**A, B, C, D**). Skin explants were infected with HSV1-Che at 1 × 10^7^ pfu/filter paper (**E, F, G**) or mock-treated (**H, I, J**), stained with hematoxylin and eosin (**E,H**), and labeled for pan-cytokeratin (**F, I**) or the HSV-1 single-strand DNA binding protein ICP8 (**G, J**). Scale bar: 50 µm.

We also analyzed hematoxylin and eosin-stained cross-sections from uninfected skin explants either mock-treated (Fig. 5A and B) or after depilation (Fig. 5C and D) that we had processed immediately (Fig. 5A and C) or after 48 hours of *ex vivo* culture (Fig. 5B and D). From the beginning of the experiments, we observed mild focal epithelial hyperplasia, mild multifocal orthokeratotic, and inconsistent parakeratotic hyperkeratosis, accompanied by mild, multifocal pigmentary incontinence with occasional presence of inflammatory cells in the superficial dermal layer, both after mock treatment (Fig. 5A) or after depilation (Fig. 5C). Based on their morphology, the inflammatory cells might have been macrophages or lymphocytes. After 48 hours of *ex vivo* culture, hyperkeratosis, epithelial hyperplasia, and pigmentary incontinence were still visible, along with few necrotic epithelial cells (Fig. 5B, arrow). The samples after depilation and 48-hour *ex vivo* culture (Fig. 5D) looked similar to the mock-treated ones after 48-hour *ex vivo* culture (Fig. 5B). There were a few necrotic epithelial cells (Fig. 5D, arrows); a mild, diffuse dermal edema; as well as inflammatory cells within the superficial dermis after depilation and 48-hour *ex vivo* culture (Fig. 5D). Based on their morphology, the inflammatory cells were interpreted as lymphocytes, macrophages, and plasma cells.

The *ex vivo* infection with HSV1-Che for 48 hours led to a marked, multifocal epithelial hyperplasia with epithelial degeneration and intranuclear inclusion bodies as well as mild, multifocal, orthokeratotic hyperkeratosis (compare Fig. 5E to the uninfected control in Fig. 5H). Immunolabeling for pan-cytokeratin confirmed an increased number of epithelial cell layers, interpreted as epithelial hyperplasia (compare Fig. 5F to the uninfected control in Fig. 5I). The HSV-1 single-strand DNA-binding protein ICP8 (53) was exclusively detected in the nuclei of infected keratinocytes, but not in any dermal cells (Fig. 5G), and also not in uninfected explant cultures (Fig. 5J).

### Characterization of dual-color HSV-1 strains

**Fig 6:**
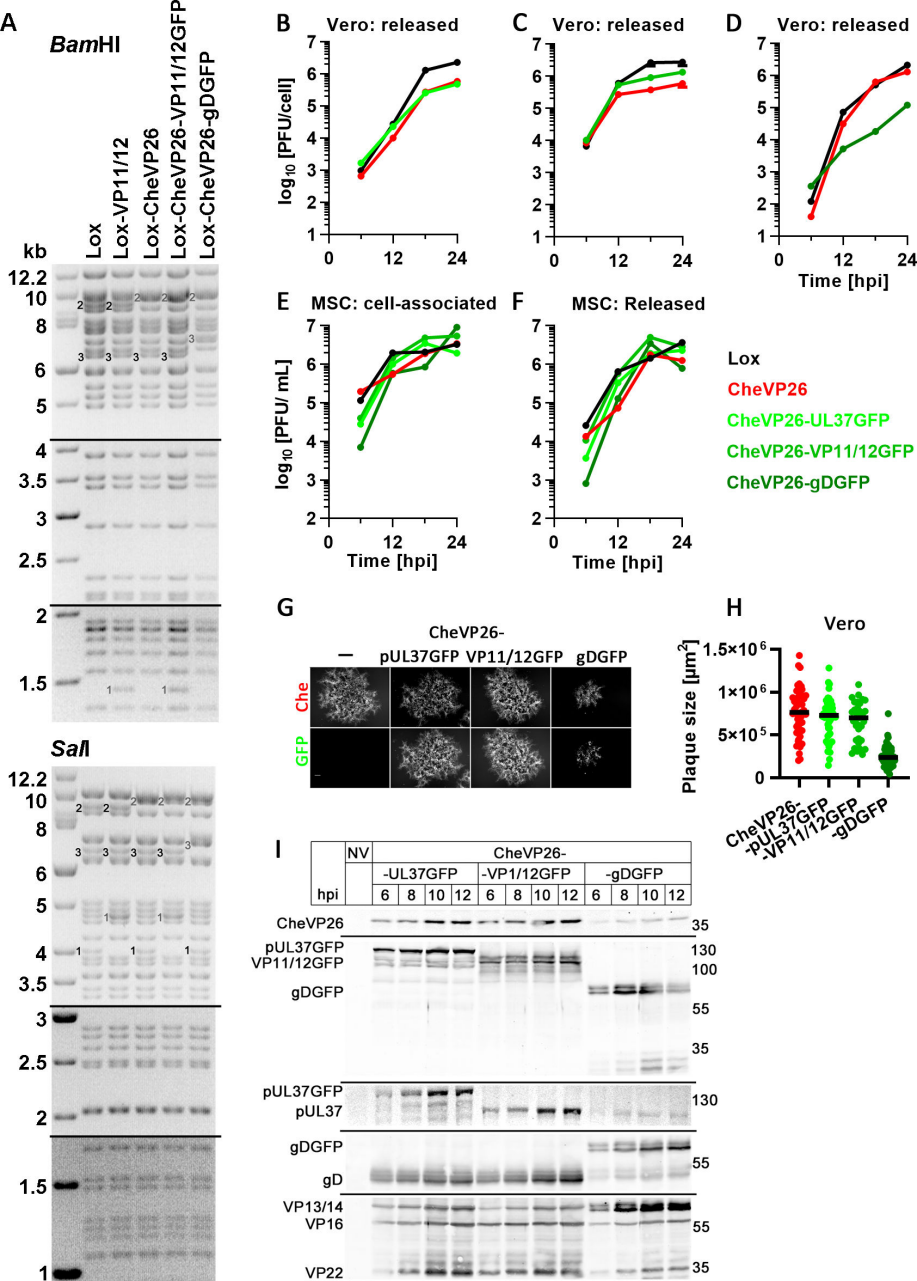
Characterization of the dual-color HSV-1 strains. (**A**) Agarose gel of genome restriction digests with *BamH*1 or Sal1 of HSV1(17^+^)Lox, Lox-VP11/12GFP, Lox-CheVP26, Lox-CheVP26-VP11/12GFP[Wi], and Lox-CheVP26-gDGFP. The fragments had shifted as expected because of adding mGFP to VP11/12, mCherry to VP26, or mGFP to gD. The sizes of the molecular weight marker bands are indicated in kB. The black numbers indicate the parental forms and gray numbers the tagged forms. (**B–F**) For single-step growth curves, Vero (**B–D**) or MSC (**E and F**) cells were infected with HSV1(17^+^)Lox (black in B–F), Lox-CheVP26 (red in B–F), Lox-CheVP26-pUL37GFP (bright green in B, **E, F**), Lox-CheVP26-VP11/12GFP[Wi] (green in C, **E, F**), or Lox-CheVP26-gDGFP (dark green in D, **E, F**) at 5 pfu/ cell (**B–D**) or 10 pfu/cell (**E and F**), and infectious virus released from infected cells into the medium (**B–D, F**) at the indicated time points and cell-associated virus (**E**) was titrated on Vero cells. (**G–H**) Vero cells were infected with HSV1-CheVP26, HSV1-CheVP26-CheVP26-UL37GFP, HSV1-CheVP26-CheVP26-VP11/12GFP[Wi], or HSV1-CheVP26-CheVP26-gDGFP, and at 2 dpi, the Cherry plaques were documented by live-cell imaging (**G**), and the plaques sizes were measured (**H**). (**I**) Immunoblot. Vero cells were mock-treated (NV) or infected for indicated time points at 10 pfu/cell with HSV1(17^+^)Lox-CheVP26-pUL37GFP, Lox-CheVP26-VP11/12GFP[Wi], or Lox-CheVP26-gDGFP, and cell lysates were analyzed by immunoblotting with antibodies raised against RFP, GFP, pUL37, gD, or various HSV-1 antigens (Remus V).

To monitor the different stages of the HSV-1 infection cycle and to characterize the intracellular distribution of different assembly intermediates, we used strains in which the small capsid protein VP26 had been tagged at its N-terminus with mCherry (54–57) and the inner tegument protein pUL37 (54), the outer tegument protein VP11/12 (58); this study), or the envelope protein gD (this study) with GFP (Table 1). Restriction digest analyses of their genomes showed that HSV1-CheVP26-VP11/12GFP with GFP added directly to its C-terminus (not shown), HSV1-CheVP26-VP11/12GFP[Wi] with GFP inserted 10 residues upstream of its C-terminus as reported before (59), and HSV1-CheVP26-gDGFP with GFP at its C-terminus showed the expected band shifts in comparison to the respective parental strains, HSV1(17^+^)Lox or HSV1(17^+^)-CheVP26, upon digestion with BamHI (Fig. 6A) or SalI (Fig. 6A). Tagging pUL37 (light green in Fig. 6B, E and F) or tagging VP11/12 (medium green in Fig. 6C, E and F) did not impair HSV-1 replication in single-step growth curves, whereas tagging gD delayed the release of infectious virus (dark green in Fig. 6D, E and F) from Vero cells (Fig. 6B, C and D) and the amount of intracellular (Fig. 6E) and extracellular (Fig. 6F) infectious viruses produced in murine keratinocytes. Accordingly, the average plaque sizes of the HSV-1 strains with a GFP tag on pUL37 or VP11/12 were similar to that of the parental strain, whereas tagging gD considerably reduced plaque size in Vero cells (Fig. 6G and H). In SDS-PAGE, the protein bands of pUL37, VP11/12, and gD shifted to the expected apparent molecular weights upon GFP tagging, with gDGFP having a similar molecular weight as VP13/14 (Fig. 6I). Moreover, the synthesis of the HSV-1 proteins CheVP26, pUL37, VP16, and VP22 was delayed in Vero cells infected with HSV1-CheVP26-gDGFP, when compared to infections with HSV1-CheVP26-pUL37GFP or HSV1-CheVP26-VP11/12 (Fig. 6I). We did not detect any differences between HSV1-CheVP26-VP11/12GFP or HSV1-CheVP26-VP11/12GFP[Wi] in our infection experiments, indicating that neither position of the GFP tag impaired the functions of VP11/12 (data not shown).

**TABLE 1:** Published and generated HSV-1 strains used in this study

HSV-1 strain 17^+^ features	Reference or this study	
	Forward primer^*a*^	Reverse primer^*a*^
HSV-1 strain 17^+^ clinical isolate	(60, 61); GenBank NC_001806	
Lox BAC clone of clinical isolate 17^+^	(50, 54)	
Lox-Che mChe between pUL55 +pUL56	(48)	
Lox-GFP mGFP between pUL55 +pUL56	(62)	
Lox-CheVP26 mChe at N-terminus of VP26	(54)	
Lox-CheVP26-pUL37GFP C-terminal mGFP at pUL37	(54)	
Lox-CheVP26-VP11/12GFP C-terminal mGFP at VP11/12	CCCTGAGCGCCCTCCTGACCAAGCT TAAACGCGAAGGACGCCGGAGCCGG GTGAGCAAGGGCGAGGAGCT	AGGGGGAAAGGGGCGTGGTCTAGCG ACGGCAGCACGGGCGGAGGCGTTCA CTTGTACAGCTCGTCCATGC
Lox-CheVP26-VP11/12GFP[Wi] with VP11/12-Thr708-mGFP-Lys709 according to (59)	TGACCAACGACGGCCCGACCAACGTCG CCGCCCTGAGCGCCCTCCTGACC GTGAGCAAGGGCGAGGAGCT	AGCACGGGCGGAGGCGTTCACCGG CTCCGGCGTCCTTCGCGTTTAAGCTT CTTGTACAGCTCGTCCATGC
Lox-CheVP26-gDGFP C-terminal mGFP at gD	CCCACATCCGGGAAGACGACCAGCC GTCCTCGCACCAGCCCTTGTTTTAC GTGAGCAAGGGCGAGGAG	CCCAACCCCGCAGACCTGACCCCCC CGCACCCATTAAGGGGGGGTATCTA CTTGTACAGCTCGTCCATG

^*a*^ The underlined sequences indicate the binding sites in the primers for the plasmid pEP-mGFP-in.

**Fig 7:**
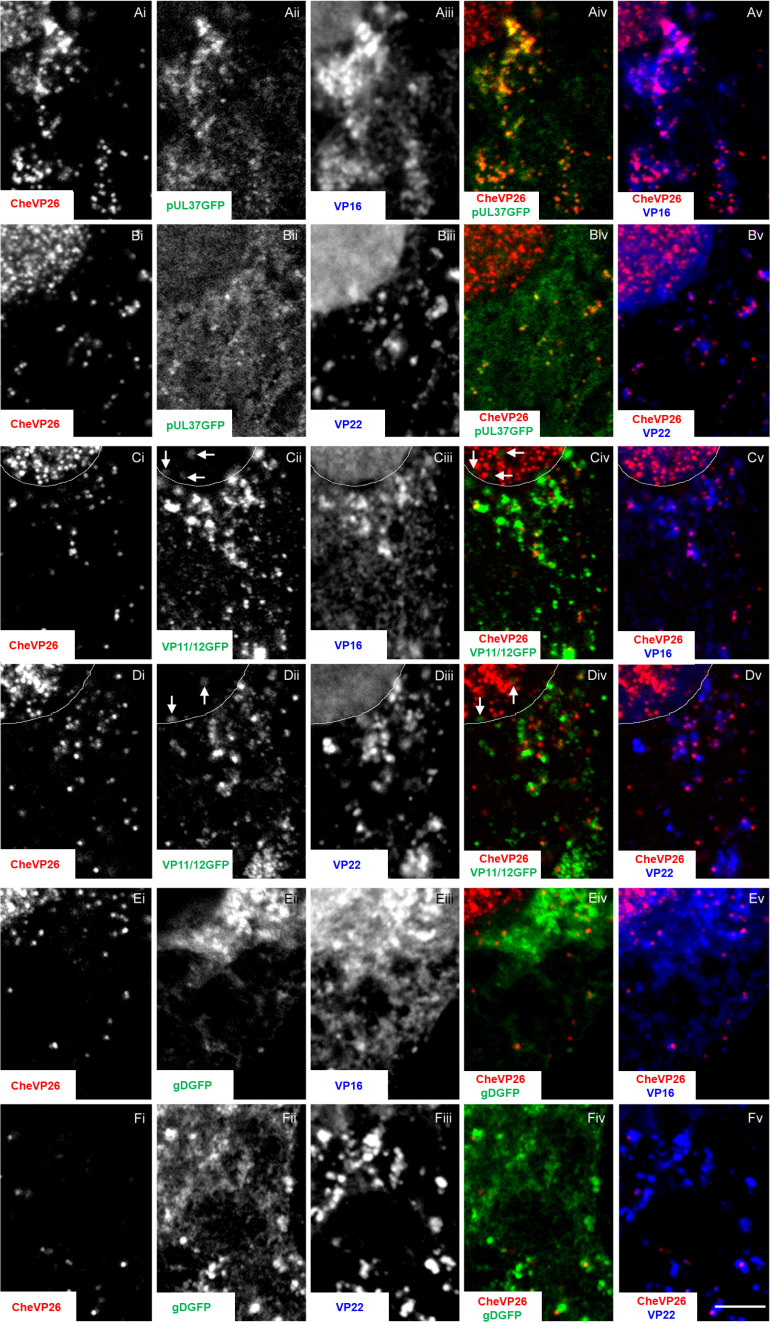
HSV-1 dual-color strains for tracking different assembly intermediates. Vero cells were infected at 10 pfu/cell (3.75 × 10^6^ pfu/mL) with HSV1-CheVP26-pUL37GFP for 10 hours (**A,B**), -CheVP26-VP11/12GFP[Wi] for 8 hours (**C,D**), or -CheVP26-gDGFP for 12 hours (**E,F**). The cells were fixed with 3% paraformaldehyde/PBS, permeabilized with 0.1% TX-100, labeled, and analyzed for HSV-1 expression of the capsid protein CheVP26 (i; red in iv and v), the inner tegument protein pUL37GFP (Aii, Bii; green in Aiv, Biv), the outer tegument protein VP11/12GFP (Cii, Dii; green in Civ, Div), the envelope protein gDGFP (Eii, Fii; green in Eiv, Fiv), VP16 (mAb LP1; Aiii, Ciii, Eiii, blue in Av, Cv, Ev), and VP22 (mAb AGV30; Biii, Diii, Fiii, blue in Bv, Dv, Fv) by confocal fluorescence microscopy. The white lines in C and D indicate the nuclear rim and arrows and Civ and Div point to nuclear VP11/12GFP dots. Scale bar: 5 µm.

Upon infection of Vero cells at a high multiplicity, all dual-color HSV-1 strains progressed relatively synchronously through virion morphogenesis, although HSV1-CheVP26-gDGFP was a bit delayed, consistent with its slower replication, plaque expansion, and HSV-1 protein synthesis (c.f. Fig. 6). All cytoplasmic but not nuclear CheVP26 capsids (Fig. 7Ai and Bi, red in Aiv, Av, Biv, and Bv) colocalized with inner tegument protein pUL37GFP (Fig. 7Aii and Bii, yellow in Aiv, Biv), while the transcriptional activators and tegument proteins HSV1-VP16 (Fig. 7Aiii, blue in Av) and VP22 (Fig. 7Biii, blue in Bv) were diffusively distributed in the nucleus and in large cytoplasmic accumulations that also contained cytoplasmic capsids, but not present on all cytoplasmic capsids. In contrast, many but not all cytoplasmic capsids (Fig. 7Ci and Di, red in Civ, Cv, Div, and Dv) localized adjacent to the outer tegument protein VP11/12GFP (Fig. 7Cii and Dii, green in Civ, Div). In addition, VP11/12GFP also localized to nuclear domains in close proximity to nuclear VP26 structures (arrows Fig. 7Cii, Dii, Civ and Div) and to the nuclear rim (not shown). Moreover, many but not all cytoplasmic capsids colocalized (Fig. 7Ei and Fi, red in Eiv, Ev, Fiv, and Fv) with the envelope protein gDGFP (Fig. 7EII, Fii green in Eiv and Fiv). While the inner tegument protein pUL37GFP could be detected on all cytoplasmic CheVP26 capsids, capsids colocalizing with the outer tegument proteins VP16 (Fig. 7AIIIiii, Ciii and Eiii; blue in Av, Cv, and Ev) and VP22 (Fig. 7Biii, Diii and Fiii; blue in Bv, Dv, and Fv) and envelope proteins were likely in the process of secondary capsid envelopment. These data suggest that the HSV-1 dual-color reporter viruses faithfully indicated the sequential acquisition of tegument proteins to capsids (reviewed in (63).

### HSV-1 capsid assembly and secondary envelopment in keratinocytes

**Fig 8:**
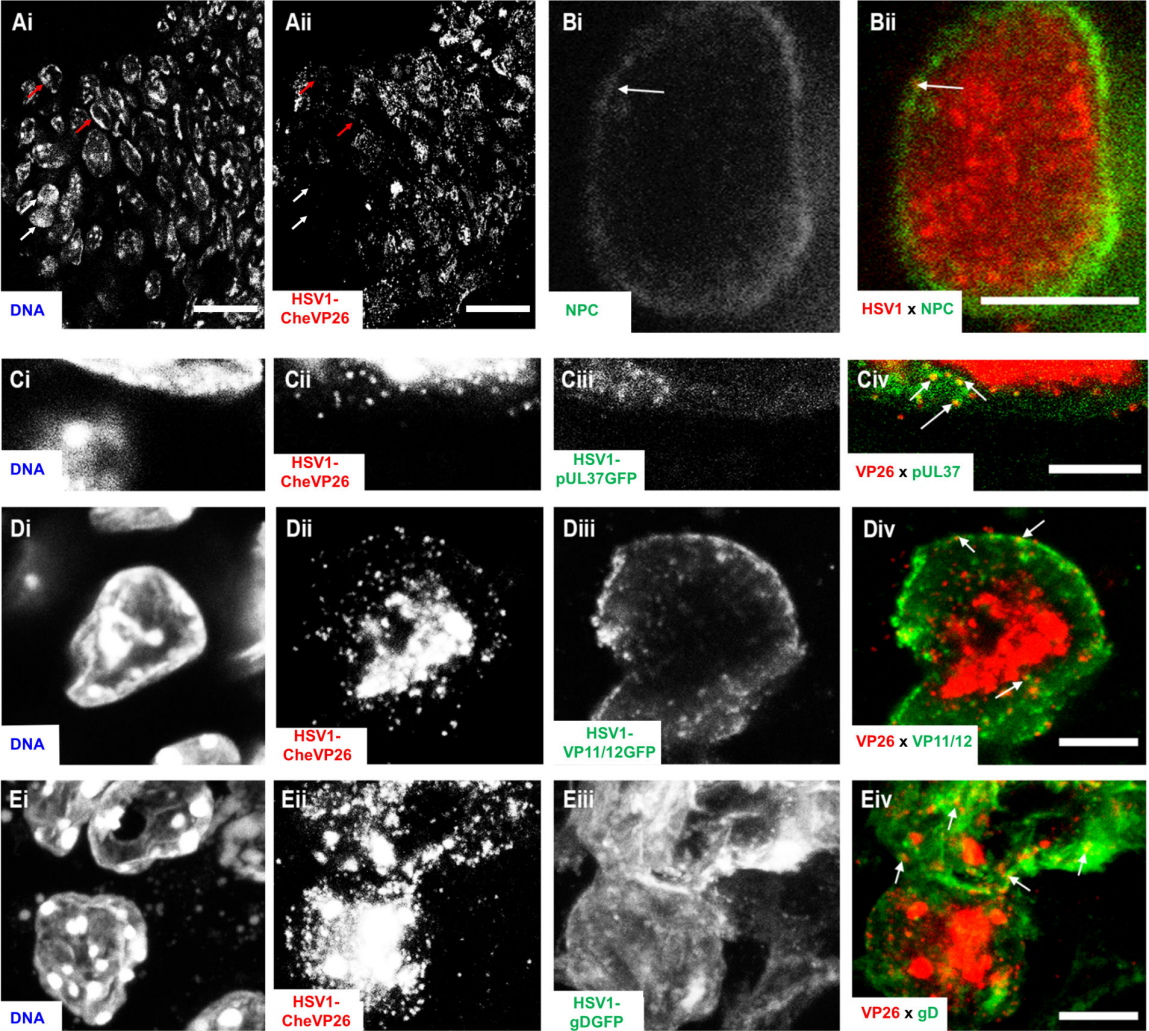
HSV-1 assembly and chromatin marginalization in murine skin keratinocytes. Ear sheets were infected with HSV1-CheVP26 (**A, B**), -CheVP26-pUL37GFP (**C**), -CheVP26-VP11/12GFP[Wi] (**D**), or -CheVP26-gDGFP (**E**) at 1 × 10^7^ pfu/filter paper for 48 hpi. Cryosections were stained with DAPI (DNA; Ai, Ci, Di, and Ei), in some cases labeled for the nuclear pores (Bi; green in Bii), and analyzed for expression of the HSV-1 capsid protein CheVP26 (Aii, Cii, Dii, and Eii; red in Bii, Civ, Div, and Eiv), the inner tegument protein pUL37GFP (Ciii; green in Civ), the outer tegument protein VP11/12GFP (Diii; green in Div), or the envelope protein gDGFP (Eiii; green in Eiv) by confocal fluorescence microscopy. Scale bars: 25 µm (**A**) or 5 µm (**B–E**).

Next, we infected skin explants with HSV1-CheVP26 to stage the infection in individual cells and monitor the formation of intracellular assembly intermediates. Confocal fluorescence microscopy of sections through infection centers cut perpendicular to the skin surface shows that the host chromatin was confined to the nuclear periphery in infected cells (red arrows in Fig. 8Ai and ii) when compared to the surrounding uninfected cells (white arrows in Fig. 8Ai and ii). Instead, the nuclei contained large accumulations of CheVP26 capsids; moreover, numerous individual CheVP26 particles were localized mainly in the nucleoplasm but also in the cytoplasm (Fig. 8Aii, Cii, Dii and Eii). Such marginalization of host chromatin also occurs during HSV-1 infection of cultured cells (64, 65). Labeling for nuclear pore proteins (Fig. 8Bi, green in Bii) indicated that the nuclear envelopes also contained capsids (white arrow in Fig. 8B). Infection with HSV1-CheVP26-pUL37GFP showed colocalization of cytoplasmic but not nuclear CheVP26 capsids with the inner tegument protein pUL37GFP (arrows in Fig. 8Civ). Similarly, after infection with HSV1-CheVP26-VP11/12GFP or HSV1-CheVP26-gDGFP, many but not all cytoplasmic capsids localized adjacent to the outer tegument protein VP11/12GFP (arrows in Fig. 8Div) or the envelope glycoprotein gDGFP (arrows in Fig. 8Eiv), respectively. In addition to its cytoplasmic and plasma membrane localization, VP11/12GFP also localized to nuclear accumulations (data not shown).

**Fig 9:**
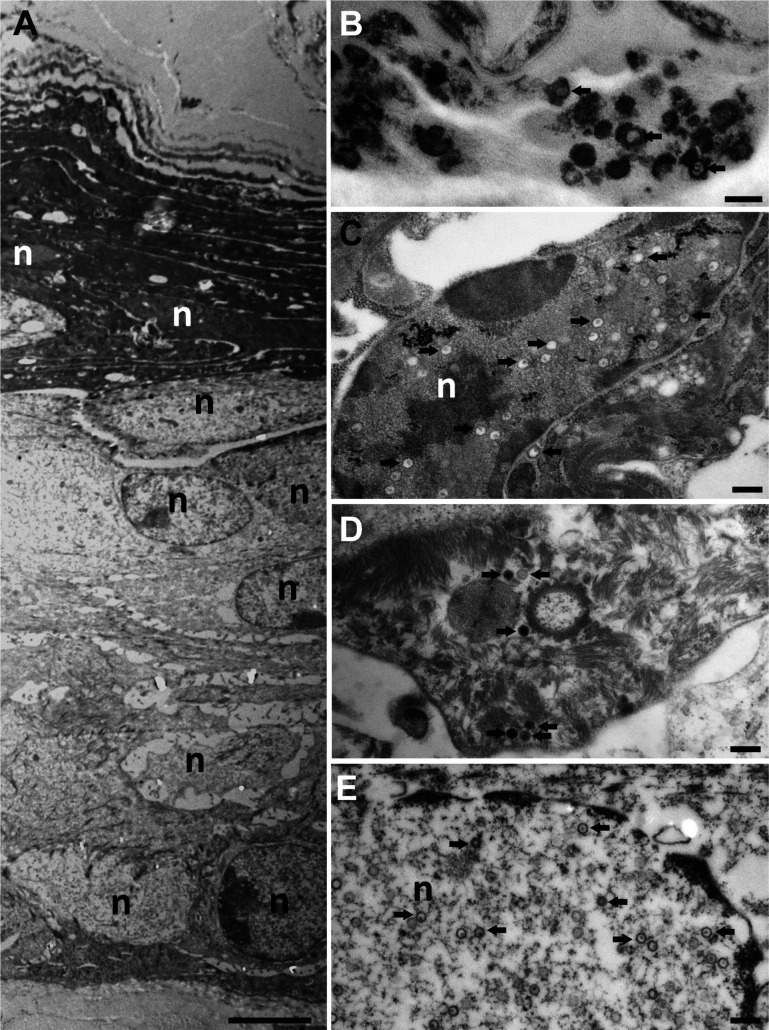
Ultrastructure of the HSV-1-infected epidermis in murine skin explants. Ear sheets were infected with HSV1-Che at 1 × 10^7^ pfu/filter paper for 48 hpi, fixed, embedded in resin, cut in ultrathin sections perpendicular to the skin surface, and analyzed by electron microscopy. (**A**) An overview at low magnification shows the four layers of the epidermis, with keratinocytes from the *stratum corneum* at the apical surface, *stratum granulosum*, and *stratum spinosum* to the *stratum basale* at the bottom of the image. (**B–D**) At higher magnifications, viral capsids (black arrows) can be recognized in all epidermis layers and extracellular viral structures in the *stratum corneum* (**B**). Keratinocytes in the *stratum granulosum* (**C**), the *stratum spinosum* (**D**), and the *stratum basale* (**E**) contained capsids in the nuclei (**C**) and the cytoplasm (**D and E**). Scale bars: 2 µm in A, 200 nm in B–E; nuclei are labeled with n.

Moreover, we analyzed the HSV-1 infection of murine skin explants by transmission electron microscopy of sections cut perpendicular through the infection centers, which we had identified by their Che fluorescence before processing for electron microscopy. The depilation had removed some but not all cells of the apical *stratum corneum,* as indicated by a remaining prominent dark keratin layer (Fig. 9A). In the *stratum corneum* that contained dead keratinocytes, we detected mostly extracellular viral structures (Fig. 9B), which probably had been released from the deeper skin layers. By electron microscopy, we detected HSV1 capsids predominantly in the nuclei and less frequently in the cytoplasm of keratinocytes in the *stratum granulosum* (Fig. 9C), the *stratum spinosum* (Fig. 9C and D), and the *stratum basale* (Fig. 9E).

**Fig 10:**
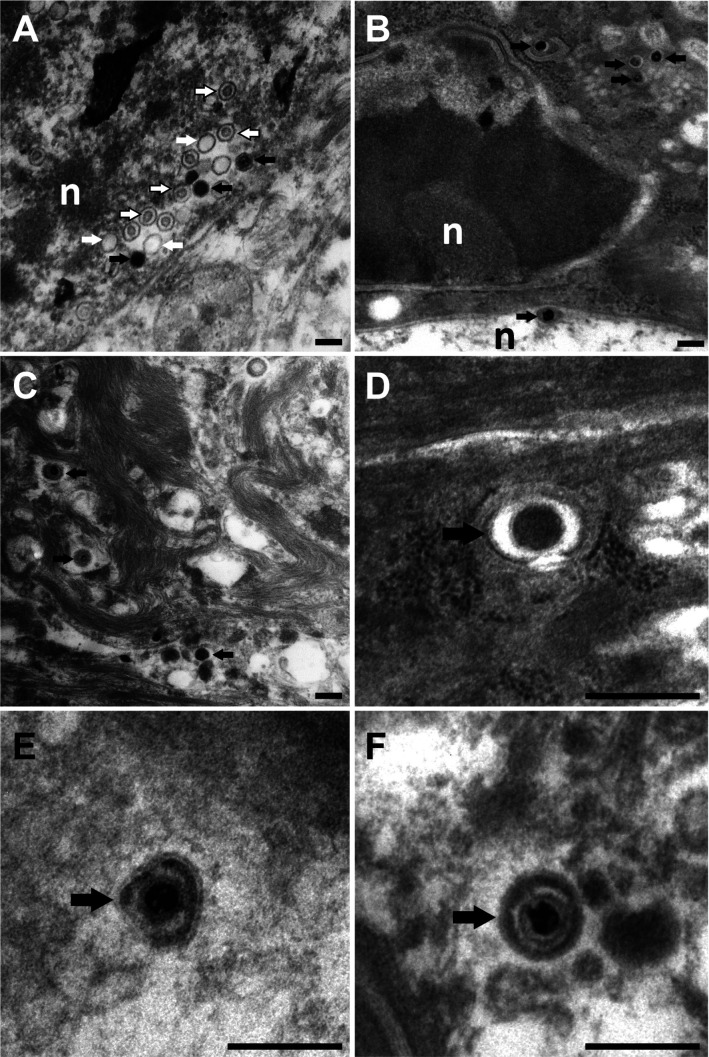
HSV-1 capsid assembly, secondary envelopment, and virion production in keratinocytes. Ear sheets were infected with HSV1-Che at 1 × 10^7^ pfu/filter paper for 48 hpi and processed for electron microscopy. The infected cells contain all HSV-1 assembly intermediates, namely, A capsids (white arrows in A), B capsids (outlined arrows in A), C capsids (black arrows in A), primary enveloped virions (black arrows in B) in the lumen between the inner and outer nuclear membrane characterized by attached ribosomes , cytosolic capsids (black arrows in C), capsids in the process of secondary envelopment (black arrow in D and E), and intracellular vesicles harboring apparently intact virions (black arrow in F). Scale bar: 200 nm; nuclei are labeled with n.

The nuclei contained capsid assembly intermediates such as empty A capsids (Fig. 10A, white arrows), B capsids with an internal protein core (outlined white arrows), and C capsids containing viral genomes (black arrows). Furthermore, we detected all known HSV-1 egress stages, namely, primary virions in the perinuclear space (Fig. 10B), cytosolic capsids (Fig. 10C), capsids in the process of secondary envelopment (Fig. 10D and E), and intracellular vesicles harboring intact virions (Fig. 10F). These results indicate efficient nuclear capsid egress and virion assembly. In summary, the fluorescence and electron microscopy data indicate that HSV-1 had productively infected the keratinocytes, that all HSV-1 assembly intermediates had been formed, and that cytosolic capsids had recruited the inner tegument protein pUL37GFP and the outer tegument protein VP11/12GFP and undergone secondary envelopment on cytoplasmic membranes containing gDGFP.

## DISCUSSION

Intact skin and mucosal barriers are essential for survival, but many viruses replicate in barrier tissues, and local immune responses often fail to provide sterilizing immunity. To better understand virus spread within barrier tissues, we established a novel murine infection model to directly observe virus-infected cells within the first few hours of infection. We inoculated from the apical outside to investigate the very early events of HSV-1 infection before the onset of clinical symptoms and without the induction of prior inflammation. HSV-1 and HSV-2 can cause painful skin lesions such as *herpetic whitlow* (“whitlow finger”) in healthcare workers (66), outbreaks of *herpes gladiatorum* (“mat herpes”) in contact-sports athletes (67), early-onset infection after cosmetic tattooing (68), and *eczema herpeticum* in a subgroup of patients with atopic dermatitis (12).

Although many small mammals can be infected with HSV-1 via several routes, murine models are favored due to the vast variety of host-specific markers and transgenic knock-in and knock-out strains (28, 29, 31). While many studies focus on the skin of the flank or the footpad (20, 38–40, 69), we infected the ear skin of 6- to 9-week-old mice because these tissue samples are very well-suited for different imaging modalities. In the long run, our approach will likely be amenable to multi-photon fluorescence microscopy analyses of cutaneous HSV-1 spread in living *ex vivo* tissues and even *in vivo* in animals. Infection of the ear pinnae of several murine strains with different HSV-1 strains mimics many aspects of human pathogenesis as it leads to acute skin lesions, infiltration of immune cells, establishment of latency in sensory neurons of the cervical ganglia, and zosteriform spread back to the skin (45, 70–72).

Instead of the commonly used skin scarification by sandpaper, needling, tattooing, or tape stripping, all of which can induce innate immune responses before infection (34, 36, 37, 73, 74), we used a short depilation treatment to allow HSV-1 access to living apical keratinocytes while leaving the dermal–epidermal junction intact. Our histology analyses revealed partially inflammatory changes both with and without depilation; however, they were slightly more pronounced after treatment. Tape stripping or depilatory cream has also been used to investigate HSV-1 infection of gamma-delta T cells and Langerhans cells in murine ear explant cultures (45). The thioglycolate acids in depilatory creams reduce inter- and intra-protein disulfide bonds; hairs break off above their roots and are removed along with the intercellular lipids and some superficial dead corneocytes (75). Further important factors for our study were using high-titer HSV-1 inocula of high quality with low particle/pfu ratios (52) and limiting their application to the areas below the filter papers.

Although the air–liquid interface skin cultures were fed only with culture medium and no longer by the blood circulation, we could detect single HSV-1-infected keratinocytes as early as 12 hpi and monitor the cutaneous spread and the development of infectious centers for up to 96 hpi using reporter strains expressing fluorescent proteins. The increasing size of the infection centers over time indicates HSV-1 cell-to-cell spread (c.f. Fig. 3K), but their increasing number also suggests the formation of additional secondary infection centers (c.f. Fig. 3G and J). Similar to experiments with murine epidermal sheets but not with cornified skin from the tails (41–43)*,* the ear skin was also more susceptible to primary HSV-1 infection from the outside after corneocytes had been removed by depilation.

However, Rahn et al. (42) could not infect keratinocytes of murine tail skin explants even after wounding or removal of the *stratum corneum*, which has been attributed to additional barriers like tight junctions in the lower keratinocyte layers (21, 22). Moreover, while it is difficult to estimate the amount of infectious HSV-1 units transmitted from the filter papers to the skin surface in our protocol, our multiplicity of infection might have been higher than that of Rahn et al. (42). In addition, their inocula might have contained more defective viral particles or cellular debris that, via stimulation of pattern-recognition receptors, might have induced an antiviral state in the skin and thereby increased intrinsic resistance barriers to HSV-1 infection (41–43, 76). Likely, we were able to obtain reproducible numbers of infection centers among different experiments by using inocula of high titer and high quality and applying them after depilation to a defined surface area via filter papers, which prevented their dilution with the culture medium.

Our novel *ex vivo* culture of terminally differentiated skin is suitable to characterize the phenotype of HSV-1 mutants impaired in evading innate immune responses and skin infection with varicella-zoster virus, poxvirus, papillomavirus, or others. Moreover, it should be feasible to infect the ear pinnae of animals via the small pieces of filter paper to deliver a relatively high dose of viral inocula and to study *in vivo* the contribution of locally induced interferons and cytokines, infiltrating immune cells, and the neuro-immune crosstalk to skin lesion formation and healing. Furthermore, such *ex vivo* and *in vivo* murine skin infections could be used to investigate potential novel antiviral small chemical compounds.

In addition, Cunningham and his team have established *ex vivo* HSV-1 infection of human genital foreskin explant cultures (26, 27). Infection of the inner foreskin of adults, a type II mucosal tissue with a thin *stratum corneum*, is possible with high-density microarray projections when the microneedles precoated with HSV-1 penetrate the epidermis beyond one-third of its thickness into the *stratum spinosum* (21). It might also be possible to adapt our protocol to human skin explants by use of depilatory cream, which in contrast to high-density microarray patches is available from many local pharmacies. Moreover, as pioneered by Knebel-Mörsdorf and her team (24, 77), we envision to characterize HSV-1 infection of skin explants from atopic dermatitis patients with defined candidate single-nucleotide polymorphisms, which are hypothesized to correlate with their susceptibility to *eczema herpeticum* in atopic dermatitis. We have identified RNAse7, the short form of thymic stromal lymphopoietin, and collagen XXIIIα as host proteins that contribute to the susceptibility to *eczema herpeticum* in patients with atopic dermatitis and modulate HSV-1 infection in human keratinocytes (78–81).

## MATERIALS AND METHODS

### Cells and antibodies

BHK-21 (ATCC CCL-10) and Vero cells (ATCC CCL-81) were cultured at 37°C and 5% CO_2_ in MEM supplemented with 1% non-essential amino acids (Cytogen, Wetzlar, Germany) and 10% or 7.5% fetal bovine serum (Good Forte; PAN-Biotech, Aidenbach, Germany), and the MSC cells, a murine keratinocyte line, were cultured in RMPI-1640 with 10% fetal bovine serum and passaged twice per week. In the plaque assays, we used human antibodies, including HSV-1 neutralizing antibodies, to limit plaque formation to cell-to-cell spread (Beriglobin; CSL Behring, Hattersheim am Main, Germany (52). For the immunofluorescence microscopy experiments, we used primary antibodies directed against host or HSV-1 proteins and secondary antibodies cross-adsorbed for species specificity and attached to fluorescent dyes (Table 2). To block binding of primary or secondary antibodies to the HSV-1-encoded Fc receptor of glycoproteins gE/gI, we used human sera of HSV-1-seronegative volunteers (52, 82).

**TABLE 2:** Primary and secondary antibodies

Antigen	Name	Type, species	Reference
Red fluorescent protein (RFP)	ChromoTek, 5F8	mAb, rat	Proteintech, Manchester, UK
GFP-hepatitis B virus core c1-149	H800	pAb, rabbit	(83)
HSV-1 proteins	Remus V	pAb, rabbit	(84)
HSV-1 capsid	SY4563	pAb, rabbit	(55)
HSV-1 ICP0	5H7	mAb, mouse	ab6513, Abcam, Cambridge
HSV-1 ICP8	11E2	mAb, mouse	ab20194, Abcam, Cambridge
HSV-1 VP16	LP1	mAb, mouse	(85)
HSV-1 VP22	AGV30	pAb, rabbit	(86)
HSV-1 gD	DL6	mAb, mouse	(87); SC-21719, SantaCruz, California, USA
HSV-1 pUL37	anti-pUL37	pAb, rabbit	(88)
Pan-cytokeratin	AE1/AE3	mAb, mouse	NBP2-29429, NovusBio, Colorado, USA
Keratin 14	LL002	mAb, mouse	NBP2-34270, NovusBio, Colorado, USA
Vimentin	D21H3	mAb, mouse	#5741, CST, Massachusetts
Rabbit IgG (H + L)	IRDye800CW	pAb, goat	LI-COR Biotechnology, Bad Homburg, Germany
Rat IgG	IRdye800CW	pAb, goat	LI-COR Biotechnology, Bad Homburg, Germany
Mouse IgG (H + L)	Cy5	pAb, goat	115–175-146, Dianova, Hamburg, Germany
Mouse IgG (H + L)	Alexa 647	pAb, goat	A32728, ThermoFisher, Dreieich, Germany
Rabbit IgG (H + L)	Alexa 488	pAb, goat	A32731, ThermoFisher, Dreieich, Germany
Rabbit IgG (H + L)	Alexa 633	pAb , goat	A21070, ThermoFisher, Dreieich, Germany
Rat IgG (H + L)	Alexa 647	pAb, goat	A48265, ThermoFisher, Dreieich, Germany

### Viruses and BAC mutagenesis

We used the clinical isolate HSV-1 strain 17^+^ (60, 61) and the derived BAC strains HSV1(17^+^)Lox (HSV1-Lox for short (50, 54); HSV1(17^+^)Lox-Che (HSV1-Che for short (48); and HSV1(17^+^)Lox-GFP (HSV1-GFP (62); Table 1). Furthermore, we used HSV1(17^+^)Lox-CheVP26 with a monomeric Cherry tag on the N-terminus of the small capsid protein VP26 (HSV1-CheVP26 for short) (54); HSV1(17^+^)Lox-CheVP26-pUL37GFP with additionally a monomeric GFP tag on the C-terminus of inner tegument protein pUL37 (HSV1-CheVP26-pUL37GFP (54), with monomeric GFP on the C-terminus of the outer tegument protein VP11/12 (HSV1-CheVP26-VP11/12GFP (58); this study), with monomeric GFP inserted into the C-terminal third of VP11/12 between threonine 708 and lysine 709 at 10 residues upstream of the STOP codon, as reported before (59); HSV1-CheVP26-VP11/12GFP[Wi]; this study), and HSV1(17^+^)Lox-CheVP26-gDGFP with monomeric GFP on the C-terminus of envelope protein gD (HSV1-CheVP26-gDGFP; this study).

To generate the novel dual-color HSV1(17^+^)Lox strains, we amplified PCR fragments for mGFP-tagging from the plasmid pEP-mGFP-in (54) using the appropriate forward and reverse primers (Table 1) and used established methods for en passant BAC-mutagenesis (57, 89–91). After transformation into *E. coli* GS1783 carrying the parental BAC pHSV1(17^+^)Lox-CheVP26 and two rounds of RED-mediated recombination, the integrity of the BAC genomes was evaluated by restriction digest with BamH1, Sal1 (Fig. 5), Asc1, EcoR1, EcoRV, Not1, and Xho1 (not shown). As described before, the newly generated dual-color reporter viruses were reconstituted by transfecting the BAC DNA into Vero cells (54). The insertion of coding sequences for mGFP was confirmed after reconstituting the respective HSV1(17^+^)Lox strains by sequencing 500 bp up and downstream of the respective insertion site (not shown).

Extracellular particles secreted from BHK-21 cells infected with HSV-1 at about 3 × 10^4^ PFU/mL (MOI of 0.01 PFU/cell) for 2 to 3 days until the cells that had detached from the culture flasks were harvested by ultracentrifugation. The pellets were resuspended in MNT buffer (30 mM MES, 100 mM NaCl, 20 mM Tris, pH 7.4), aliquoted, snap-frozen in liquid N_2_, and stored at −80°C in single-use aliquots, as reported before (52, 92). The stocks had a titer of 2 × 10^8^ to 2 × 10^10^ PFU/mL on Vero cells and a genome/PFU ratio of 20 to 50, indicating very high-quality inocula with a low amount of defective, interfering particles (52, 82)

### Plaque assay

The HSV-1 stocks, as well as the explant-associated virus, were titrated on Vero cells, as reported before (52, 82) The explants were removed from the filter papers, cut into small pieces of about 1 mm^3^, sheared (TissueRuptor II; Qiagen, Hilden, Germany) in MNT buffer, and freeze-thawed three times to release any cell-associated viruses. After the inoculation for 1 hour, the cells were incubated in a regular culture medium containing 400 µg/mL human antibodies to limit the spread of extracellular virions (Beriglobin; CSL Behring, Hattersheim am Main, Germany).

### Infections

C57BL/6 mice were bred at the Central Animal Facility of the Hannover Medical School. Female mice aged 6 to 9 weeks were sacrificed under anesthesia by inhalation of CO_2,_ followed by cervical dislocation according to local animal welfare regulations, the ears were cut off, washed with 70% ethanol, and treated with depilatory cream for 3 minutes (Veet PURE-hair removal crème for sensitive skin; Reckitt Benckiser Deutschland GmbH, Heidelberg, Germany), and the hairs and the cream were gently removed with cotton buds and PBS. S-shaped tweezers were used to separate the ears into apical and basal sheets. The sheets were glued with tissue adhesive (Surgibond, SMI AG, St. Vith, Belgium) onto slightly larger pieces of tissue gaze, which were placed into 6- or 12-well plates containing CO_2_-independent culture medium (Gibco, Thermo Fisher Scientific, Dreieich, Germany) supplemented with 0.1% [wt/vol] cell-culture grade BSA (Sigma Aldrich, St. Louis, Missouri) for about 10 minutes on ice. For inoculation, the medium was removed, a sterile piece of filter paper of 5 mm diameter was placed onto each skin sheet, and 10 µL of MNT buffer without or with varying amounts of HSV-1 was pipetted into the center of a given filter paper. After 30 minutes at 37°C and 5% CO_2_, the filter papers were removed, and 1 mL/well of DMEM supplemented with 10% [vol/vol] FBS, 100 IU/mL penicillin, 100 µg/mL streptomycin, and 1 µg/mL amphotericin B was added. The skin explants were incubated at 37°C and 5% CO_2_ for the indicated times and fixed with 3% paraformaldehyde in PBS at RT for 24 hours. For quality control, images of the skin explants were documented with wide-field fluorescence microscopy (c.f. Fig. 1; ZEISS observer Z1, Carl Zeiss Microscopy GmbH Jena, Germany; CMOS camera).

For synchronous HSV-1 infections of cultured cells, Vero cells were pre-cooled for 20 minutes on ice and inoculated with an MOI of 10 PFU per cell or mock-treated as a control in CO_2_-independent medium containing 0.1% (wt/vol) BSA for 2 hours on ice while rocking. The cells were then shifted to regular growth medium at 37°C and 5% CO_2_ for 1 hour. Any non-internalized virus was inactivated by a 3-min acid wash (40 mM citrate, 135 mM NaCl, 10 mM KCl, pH 3) at 4°C, and after washing, cells were further incubated in Vero medium for indicated times.

### Detection of host and HSV-1 genomes and transcripts

Infected skin pieces were removed from the filter papers, cut into small pieces of about 1 mm^3^, and sheared (TissueRuptor II; Qiagen, Hilden, Germany). We used the QIAamp DNA Mini and Blood Mini kits (Qiagen) to isolate the DNA and the Q5 High-Fidelity DNA Polymerase (Thermo Fisher Scientific, Dreieich, Germany) with the primers HSV-1 gB-forward (5′-gtagccgtaaaacggggaca-3′) and gB-reverse (5′-ccgacctcaagtacaacccc-3′) or murine pthrp-forward (5′-ggtatctgccctcatcgtctg-3′) and pthrp-reverse (5′-cgtttcttcctccaccatctg-3′) at an annealing temperature of 60°C to measure the amount of HSV-1 genomes relative to the host genomes. We used TRIzol (Thermo Fischer Scientific, Dreieich, Germany) to isolate the RNA and LunaScript RT SuperMix to produce the cDNA. The cDNA was amplified with Phusion high-fidelity DNA polymerase (Thermo Fisher Scientific, Dreieich, Germany) and the primers HSV-1 gB-forward (5′-cgcatcaagaccacctcctc-3′) and gB-reverse (5′-agcttgcgggcctcgtt-3′) or murine actin-forward (5′-ggctgtattcccctccatcg-3′) and actin-reverse (5′-ccagttggtaacaatgccatgt-3′) for amplification.

### One-step growth curves

Sub-confluent Vero or MSC cells were inoculated at a multiplicity of infection of 5 PFU per cell for 1 hour at room temperature on a rocking platform, and supernatants and the cells were harvested at the indicated time points. The cells were scraped in MNT buffer (30 mM MES, 100 mM NaCl, 20 mM Tris, pH 7.4), and virus was released by three cycles of freeze–thawing. Intra- and extracellular virus were titrated on Vero cells, as described before (52). For this, cells were inoculated with serial dilutions of the samples and incubated for 1 hour at room temperature while rocking. Afterwards, they were incubated with the growth medium containing 20 µg per mL pooled human IgGs (Sigma-Adrich, Schnelldorf, Germany). To determine the plaque size, images of living cells were acquired using a Zeiss Observer Microscope and 10 x objective with appropriate filter sets and analyzed in the FIJI software (version 1.50 a). Plaque margins were outlined manually along the line where the CheVP26 fluorescence intensity dropped using the “freehand selection” tool. The area of the plaque was determined using the measurement function of the FIJI software. To determine the number of plaques, the cells were fixed in absolute methanol 3 days later and stained with 0.1% (wt/vol) crystal violet.

### Immunoblot

Cells were washed with PBS and lysed with hot sample buffer (50 mM Tris-HCl, pH 6.8, 1% [wt/vol] SDS, 1% [vol/vol] β-mercaptoethanol, 5% [vol/vol] glycerol, and 0.001% [wt/vol] bromophenol blue) containing protease inhibitors AEL (aprotinin, E-64, leupeptin, Sigma), ABP (antipain, bestatin, and pepstatin, Sigma), and PMSF (phenylmethylsulfonylfluorid in isopropanol, Roth, Karlsruhe, Germany). Cell lysates were boiled at 95°C for 5 minutes and homogenized by needling. The proteins were separated by 7.5%–18% SDS-PAGE and transferred in 24 mM Tris, 193 mM glycine, 0.035% [wt/vol] SDS and 15% [vol/vol] methanol to nitrocellulose membranes (Pall Corporation, Pensacola, FL, USA). After blocking with 5% [wt/vol] low-fat milk powder in PBS containing 0.1% [vol/vol] Tween 20 (PBS-T), the membranes were probed with primary antibodies and secondary antibodies coupled to fluorescent dyes (Table 2; LI-COR Biotechnology, Bad Homburg, Germany) and documented with line scanning and a digital image sensor (Odyssey Infrared Imaging System, Li-COR Biosciences, NE, USA).

### Confocal fluorescence microscopy

Cultured cells were fixed at room temperature with 3% [wt/vol] paraformaldehyde for 20 minutes and permeabilized with 0.1% Triton-X-100 for 5 minutes or treated at 37°C with 3.7% [wt/vol] paraformaldehyde, 0.05% [wt/vol] glutaraldehyde, 0.5% Triton-X-100 in PHEMO buffer with 68 mM PIPES, 25 mM HEPES, pH 6.9, 15 mM EGTA, 3 mM MgCl_2_, and 10% dimethyl sulfoxide (93). After either fixation protocol, the specimens were incubated with 50 mM NH_4_Cl for 10 minutes to inactivate any remaining paraformaldehyde and glutaraldehyde. The HSV1 Fc-receptor and unspecific protein binding sites were blocked in blocking buffer (10% [vol/vol] human serum of an HSV-1 seronegative volunteer, 0.5% [wt/vol] BSA, in PBS, pH 7.4). The samples were labeled with primary and pre-adsorbed secondary antibodies to prevent species cross-reactivity (Table 2). The cover slips were mounted in Mowiol 4–88 containing 2.5% (wt/vol) 1,4-diazabicyclo-[2.2.2]octane. Experiments were analyzed using a confocal fluorescence microscope equipped with a 63 x objective (TCS SP8, LEICA Microsystems, Wetzlar, Germany).

For immunolabeling of cryosections, the ear skin explants were fixed at 4°C with 3% paraformaldehyde in PBS overnight, treated with 50 mM NH_4_Cl in PBS for 30 minutes to quench residual paraformaldehyde, and infiltrated with 30% [wt/vol] sucrose in PBS overnight at 4°C. After embedding on dry ice in O.C.T. compound (Sakura Finetek Europe B.V., Umkirch, Germany), the specimens were cut into 8- to 20-µm-thick sections (Leica CM3050 S cryostat, Wetzlar, Germany) and transferred to adhesive glass slides (Thermo ScientificTM SuperFrost Ultra Plus GOLD, Thermo Fisher Scientific, Dreieich, Germany). The sections were incubated for 30 minutes at RT in blocking buffer with 0.3% [vol/vol] Triton-X 100, labeled with primary antibodies in blocking buffer at 4°C overnight and pre-adsorbed secondary antibodies to prevent species cross-reactivity (Table 2) in blocking buffer (10% [vol/vol] human serum of an HSV-1-seronegative volunteer, 0.5% [wt/vol] BSA, in PBS, pH 7.4) at RT for 90 minutes, and mounted in Mowiol 4–88 containing 2.5% [wt/vol] 1,4-diazabicyclo-[2.2.2]octane. Images of optical sections were captured with a confocal microscope and a 20 x or 63 x objective (TCS SP8, LEICA Microsystems, Wetzlar, Germany).

Images of a given experiment were adjusted with identical linear contrast and brightness settings, merged, and evaluated using the Fiji image analysis platform (94)

### Two-photon fluorescence microscopy

Ear sheets were fixed in 3% paraformaldehyde in PBS at 4°C overnight and attached to the bottom of an imaging chamber with tissue adhesive. The samples were investigated at RT with two-photon imaging using two laser lines in parallel and PBS as the optical medium (LaVision Bio Tec TrimpScope 2; Miltenyi Biotec, Bielefeld, Germany). mCherry was excited with a Spectra-Physics Insight laser set to 1,105 nm, and GFP and dermal collagen were visualized by a second laser set to 910 nm at an intensity of 1% to 10% of its maximum power. The emitted light was captured with a 20 x objective with a standard voxel size of 4 µm^3^ (NA 0.9; ZEISS) and recorded with photononic multiplier tubes (Hamamatsu) with different bandpass filters, namely, a blue channel at 400–450 nm for the dermal collagen, a green channel at 510–550 nm for GFP, and a red channel at 610–640 nm for mCherry. Images were acquired with the LaVision Inspector Software Version 3 and analyzed with the Imaris software (Version 9.1; Bitplane at Oxford Instruments; Oxfordshire, UK) as reported before (95).

### Histopathology and immunohistochemistry

Ear sheets were fixed in 3% paraformaldehyde in PBS overnight at 4°C, embedded in paraffin after trimming of cross-sections, and cut into 5-µm-thick sections. For histopathological examination, sections were stained with hematoxylin and eosin. The severity of histopathological lesions was graded into (i) mild epithelial hyperplasia with up to 5 layers of epithelial cells, (ii) moderate epithelial hyperplasia with 6 to 10 layers of epithelial cells, (iii) marked epithelial hyperplasia with more than 10 layers of epithelial cells, (iv) mild pigmentary incontinence with 1 to 5 melanophages per high-power field (HPF), (v) moderate pigmentary incontinence with 6 to 10 melanophages per HPF, or (vi) marked pigmentary incontinence with more than 10 melanophages per HPF.

Immunohistochemistry was performed as reported before (96). Briefly, the sections were deparaffinized and rehydrated, and endogenous peroxidase activities were inhibited. For antigen retrieval, the sections were incubated with citrate buffer (pH 6.0) in the microwave oven at 800 W for 20 minutes. The primary antibodies pan-cytokeratin AE1/AE3 and anti-HSV1-ICP8 were applied in PBS with 1% bovine serum albumin overnight at 4°C. Negative controls were generated by substituting primary antibodies with Balb/c mouse ascites fluid (diluted 1:1,000 in PBS). The sections were incubated with EnVision +System HRP-labeled polymer (Dako Agilent Pathology Solutions, Agilent Technologies Deutschland GmbH, Waldbronn, Germany) for 30 minutes at RT. The immunoreactions were visualized by adding 3,3’-diaminobenzidine tetrahydrochloride (0.05%, Sigma Aldrich Chemie GmbH, Darmstadt, Germany) with 0.03% hydrogen peroxide for 5 minutes at RT, and the sections were counterstained with Mayer´s hematoxylin (Roth C. GmbH & Co KG, Karlsruhe, Germany).

### Electron microscopy

Ear sheets were infected with HSV1-Che at 1 × 10^7^ PFU per filter paper for 48 hours. The samples were fixed in 2% [wt/vol] glutaraldehyde, 2.5% [wt/vol] paraformaldehyde, 165 mM sodium cacodylate, pH 7.4, 2 mM CaCl_2_, and 10 mM MgCl_2_ at RT for 1 hour. To locate the small infection centers, the explants were placed with the apical surface onto coverslips with a gridded checkerboard of alphanumeric patterns (P35G-2–14-C; MatTek Europe, Slovak Republic) and embedded in 10% [wt/vol] gelatin in PBS at 37°C. The coverslips were immediately placed on an ice-cold metal plate to solidify the gelatin, and the fresh fixative was added on ice for 1 hour to crosslink the gelatin to the skin sheets. As ethanol and propylene oxide used for resin embedding destroy the fluorescence of mCherry, we localized infection centers at this point of the protocol in reference to the gridded checkerboard by their HSV-1-mediated mChe expression using a wide-field fluorescence microscope (Leica Microsystems, Germany) and the filter set N2.1 containing the emission long-pass filter LP590 which largely blocks the autofluorescence generated by the glutaraldehyde fixation. Subsequently, the specimen were contrasted with 1% [wt/vol] OsO_4_ in 165 mM cacodylate buffer, pH 7.4 containing 1.5% [wt/vol] K_3_[Fe(CN)_6_] for 1 hour, followed by 0.5% [wt/vol] uranyl acetate in 50% (vol/vol) ethanol overnight, the ethanol chain for dehydration, a propylenoxide intermediate, and embedding in expoxy resin (Epon, Serva, Heidelberg, Germany). The gridded coverslips were detached from the polymerized resin but had left their checkerboard imprints in the resin to locate the positions of the infection centers. These skin areas were carved from the resin, cut in half, and each half was glued onto a separate resin block at a 90° angle, with the cut surface facing the microtome knife to obtain cross-sections through the infected skin explants. Ultrathin sections of 50 nm thickness were cut and further contrasted using lead citrate (97) and uranyl acetate (98). Images were taken at 80 kV with a transmission electron microscope (Morgani, FEI, The Netherlands) equipped with a digital camera (Veleta; Olympus Soft Imaging Solutions, Germany) and processed using the Fiji image analysis platform (94)

### Statistical analyses

Data were presented in scatter plots with lines at the median with the median interquartile range as error bars. We performed Wilcoxon signed-rank, Mann–Whitney U, and *t*-tests (GraphPad Prism v5.0; La Jolla, California) and calculated the standardized effect sizes and the statistical power ([99]; Gpower 3.1; https://gpower.software.informer.com/3.1/). A *P*-value of less than 0.05 was considered significant.
